# Variations in Crystalline Structures and Electrical Properties of Single Crystalline Boron Nitride Nanosheets

**DOI:** 10.1038/srep16703

**Published:** 2015-11-13

**Authors:** Ali Aldalbahi, Andrew Feng Zhou, Peter Feng

**Affiliations:** 1 Department of Chemistry, King Saud University, Riyadh 11451, Saudi Arabia; 2 Department of Physics, Indiana University of Pennsylvania, Indiana, PA 15705, USA; 3 Department of Physics, University of Puerto Rico, San Juan, PR/USA 00936-8377

## Abstract

We report the studies of (1) the basic mechanism underlying the formation of defect-free, single crystalline boron nitride nanosheets (BNNSs) synthesized using pulsed laser plasma deposition (PLPD) technique, (2) the variation in the crystalline structure at the edges of the hexagonal boron nitride (h-BN) nanosheets, and (3) the basic electrical properties related to the BNNSs tunneling effect and electrical breakdown voltage. The nanoscale morphologies of BNNSs are characterized using scanning electron microscope (SEM) and high-resolution transmission electron microscope (HRTEM). The results show that each sample consisted of a number of transparent BNNSs that partially overlapped one another. Varying the deposition duration yielded different thicknesses of sample but did not affect the morphology, structure, and thickness of individual BNNSs pieces. Analysis of the SEM and HRTEM data revealed changes in the spatial period of the B_3_–N_3_ hexagonal structures and the interlayer distance at the edge of the BNNSs, which occurred due to the limited number of atomic layers and was confirmed further by x-ray diffraction (XRD) study. The experimental results clearly indicate that the values of the electrical conductivities of the super-thin BNNSs and the effect of temperature relied strongly on the direction of observation.

Research into two-dimensional (2D) atomic-thin sheets has brought about a new revolution in multifunctional materials science because of many attractive but unusual properties of these 2D nanosheets^1^,^2^,^3^,^4^,^5^. A typical example of such materials is atomic thin single-layer boron nitride. Recent studies have shown that 2D boron nitride nanosheets (BNNSs) possess enormous potential for various applications, including uses in thermal conductivity, spintronics, and tissue engineering, and as dielectric coatings with excellent heat dissipation properties (thermal paste), nanoscale supports for metal or metal oxide catalysts, thermal radiators, deep ultraviolet light emitters, drug delivery platforms and labels, and critical components in nanodevices^6^,^7^. The lattice constant is nearly identical to that of graphene, which suggests that BNNSs can be combined with graphene to form single-molecule circuits. BNNSs may also be used to inhibit oxidation in graphitic materials.

Many studies in synthesis of BNNSs have been conducted by using various techniques, including plasma sputtering^8^, chemical vapor deposition (CVD)^9^,^10^, thermal vapor solid target^11^, chemical exfoliation^12^,^13^, chemical blowing^14^, ball milling^15^, micromechanical cleavage^16^, and liquid exfoliation^17^ of bulk h-BN. Over the last four years, Kim^18^, Yu^19^, Khan^20^ and many others have successfully used CVD process to synthesize large BNNSs. Recently, several important works related to atomic thin BNNSs oxidation resistance and dielectric screening have been reported in many literature^21^,^22^,^23^. Systematic and comprehensive reviews of 2D boron nitride nanostructures have also been completed^24^,^25^.

However, the reported growth rate in CVD process is extremely low^19^,^25^, and in most cases, the synthesis using CVD process could also yield certain impurities. This is because the CVD precursors often contain multiple component substances rather than a pure element^26^. Furthermore, the increase in temperature up to 1000 °C in CVD^20^,^25^ process would not only vaporize the impurities inside the chamber but also result in internal stresses that may affect the crystalline structures of the BNNSs.

Using the pulsed plasma beam deposition technique, we have conducted several experiments to synthesize free-standing BNNSs^27^,^28^. In the present paper, we have reported our new studies of crystalline lattice structure variations, temperature effects, zigzag edge reconstructions, the quantum tunneling effect and the basic mechanism of the formation of defect-free, single crystalline BNNSs.

## Experimental Conditions

A CO_2_ pulsed laser plasma deposition (PLPD) technique was used to synthesize BNNSs. A detailed description of the PLPD system can be found in our previous published article^29^. Briefly, the laser beam at 10.6 μm wavelength, was focused with a 30-cm focal length ZnSe lens, incident at 45 degree relative to a rotating (speed of ~200 rpm) pyrolytic hexagonal BN target (2.00″ diameter × 0.125″ thickness, 99.99% purity, B/N ratio ~1.05, density ~1.94 g/cc) under high vacuum (2 × 10^−3^ Pa) in an enclosed chamber. The laser operated at a repetition rate of 5–10 Hz. The laser pulse width was 2 μs, and the pulse energy was 5 J. The purpose of the use of long-focal-length lens was to effectively control the pulsed laser-produced plasma beam. The diameter of the focal spot of the laser beam on the target was approximately 2 mm. The power density of the laser on the target was 2 × 10^8^ W/cm^2^ per pulse, with which we could achieve mass production of BNNSs up to 15–50 mg per hour, depending on the repetition rate of laser operation. An increase in power density would have yielded more BNNSs, but the quality of the BNNSs would have been slightly different. Molybdenum (Mo), ceramic aluminium nitride (AlN), and quartz were used as substrates and placed 5 cm away from the target. Prior to laser irradiation, the substrates were rinsed with acetone and methanol in a sequence. The duration for each deposition was 10 seconds, corresponding to 50 ~ 100 pulsed plasma beams for sample synthesis. The BNNSs were then characterized by scanning electron microscope (SEM), transmission electron microscope (TEM), Raman scattering spectroscopy, x-ray diffraction (XRD) study, and electrometry.

## Results and Discussions

### Crystalline structures

**Figure 1:**
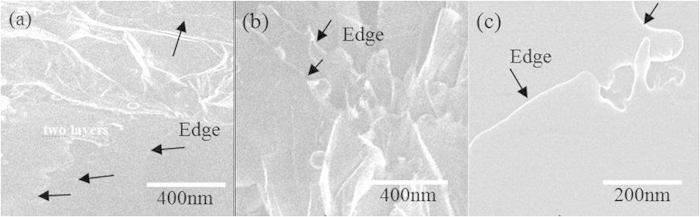
SEM images of BNNSs prepared at different substrate temperatures: (**a**) 450 °C, (**b**) 350 °C, and (**c**) 250 °C.

Figure 1 shows typical SEM images of the BNNSs that were prepared at different substrate temperatures. Each sample consisted of several sheets partially overlapping one another. The sample surfaces possessed different morphologies. It was found that 350 °C is the critical substrate temperature in the present synthesis of high-quality BNNSs. For example, synthesis at 450 °C substrate temperature yielded highly transparent BNNSs (Fig. 1a), and this property is related to the high quality of the crystalline structure. The average thickness of the BNNSs was 2.5 nm, and the average size was larger than 2 μm^2^. These values were obtained from statistical data using high-resolution TEM and SEM. We have found that by controlling the gas and pressure environment inside the chamber, we can control the thickness of BNNSs. For example, the average thicknesses of the BNNSs that were synthesized in hydrogen and in high vacuum environments were very different. The size of the BNNSs was affected by the pulse of the laser-produced plasma beam (e.g., pulse width, beam intensity, the laser focal spot size on the target). The mechanism is complicated because it involves plasma dynamic processes. Several experiments have been conducted to study the effects of the laser plasma beam on the size, but the exact relationship has not yet been concluded. Synthesis of BNNSs at 250 °C or lower yielded opaque films, as shown in Fig. 1c. Consequently, it was impossible to obtain sharp XRD peaks from the sample. This phenomenon demonstrates that heat provided additional energy to promote molecular migrations, which led to a crystalline structure. The results suggest that it would not be possible to synthesize single crystalline BNNSs below a certain temperature.

**Figure 2:**
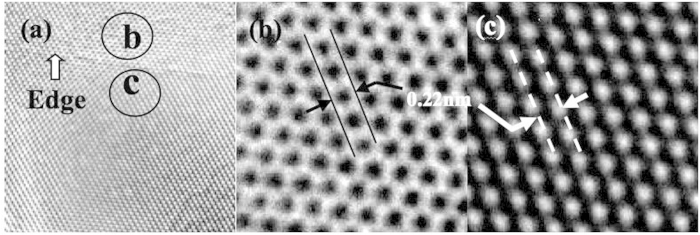
(**a**) Typical HRTEM image and (**b,c**) further magnified HRTEM images of selected areas (**b**) and (**c**) of the BNNSs .

High transparency related to high quality in BNNSs was confirmed with a high-resolution TEM (HRTEM) microscope equipped with an aberration corrector. Figure 2 shows the HRTEM studies of BNNSs at different magnifications. The shaped edges of the surface of a single BNNS (Fig. 2a) could be easily identified, which indicated that single crystalline BNNS actually consisted of a few atomic layers. Clearly, each atomic layer consisted of a large amount of highly ordered boron and nitrogen atomic arrays.

Figure 2b shows an enlarged image of the selected area (b) from Fig. 2a. Honeycomb structures of six-membered B_3_–N_3_ hexagons are clearly visible. The spatial period was approximately 0.22 nm, as shown in Fig. 2b. Within an atomic layer, B and N atoms were bound strongly by covalent bonds. Neither N−N nor B−B bonds were observed in the characterized area, which signified the single crystalline structure of the synthesized BNNS. Figure 2c shows the HRTEM image of another selected area (c) from Fig. 2a. Similarly, there were neither defects nor impurities over the entire examined area (c). HRTEM images with slight aberrations were observed, but highly ordered arrays with a 0.22 nm period were still noticeable.

**Figure 3:**
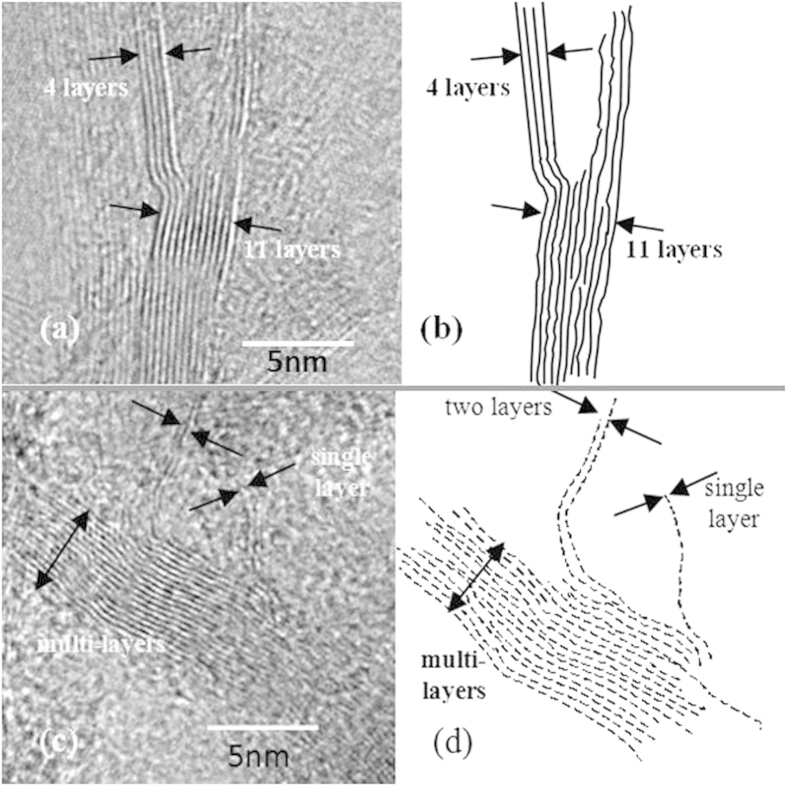
TEM images of structures (**a,c**) for different edges of a BNNS sample and (**b,d**) the models for mono-, bi-, and multi-layer structures of a BNNS.

The mechanism for the formation of crystalline BNNSs relies heavily on the selected method of synthesis. In the present case, we believe that heat-driven mechanical exfoliation should dominate as the main process in the formation of crystalline BNNS. Two solid pieces of evidence have been found. Figure 3a shows a typical HRTEM image of the structure at different edges of a BNNS sample. The model for the multi-layer structure is depicted in Fig. 3b. Each fringe is related to a single atomic layer, which has a thickness of approximately 0.33 nm. Each sheet consists of multiple layers, and the single sheet with 11 atomic thin layers splits partially into two portions. Such a phenomenon has not been reported in the case of a BNNS that was grown using CVD technique. It was believed due to a different crystal structure formation mechanism which was a combination of epitaxial growth from the substrate in the beginning and followed by a diffusion-segregation process^30^.

Figure 3c shows another HRTEM image of the structure at the edge of a BNNS sample. The model for mono-, bi-, and multi-layer structures shown in Fig. 3d strongly suggests that the basic mechanism for the formation of 2D BNNS is related to the thermo-mechanical exfoliation of bulk BN. Because the short-pulse plasma beam used in the present deposition is in the one-way drift, the interaction of intense short laser pulses with material targets normally leads to the anomalous and normal skin effects in plasma with step-like density profile, whereby the heat transport can be described by classical Spitzer conductivity, with the new boundary conditions accounting for laser absorption in the thin skin layer^31^. The rapid heating of the thin skin layer of van der Waals solids such as h-BN would unavoidably result in thermo-mechanical exfoliation. This procedure leads to 2D growth and high transparency of BNNSs. Furthermore, BNNSs are already formed before reaching the substrate. As a result, the quality (morphology and crystalline structure) of individual continuous BNNSs would not be significantly affected by the substrate type. This phenomenon has already been observed and reported in literature^27^.

**Figure 4:**
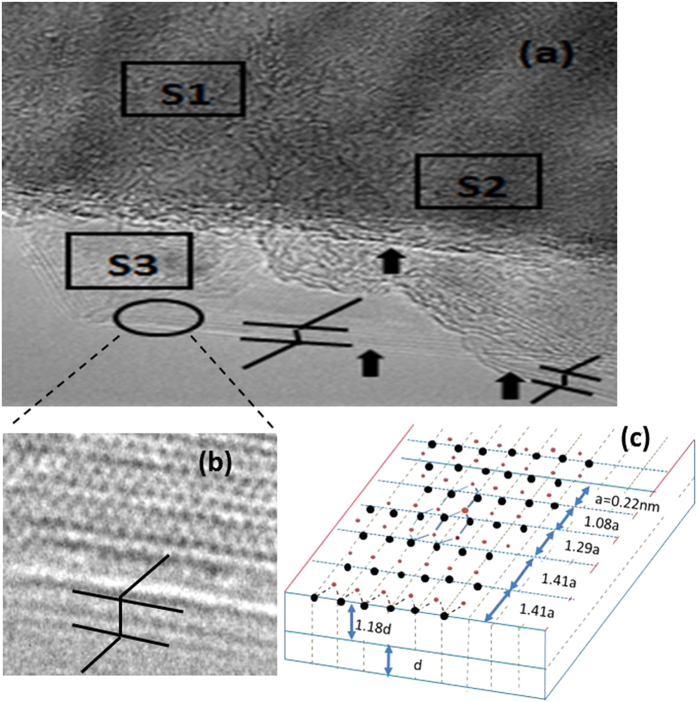
(**a**) HRTEM image of an edge area, (**b**) magnified HRTEM image of a selected area of the BNNS, and (**c**) an atomic model for the edge area.

Variations in the interlayer distance (“sort of” lattice constant) and atom-to-atom spacing are also observed at the area near the edge of the BNNS, as shown in Fig. 4a, where three partially overlapped sheets, marked S1–3, can be observed. Based on the crystalline structures shown in Figs 2b and 4b, the atomic model for the edge area is constructed as shown in Fig. 4c, from which we can conclude that the BNNS has zigzag edge reconstructions terminated by boron atoms. By contrast, it is clear that the three sheets, marked in Fig. 4a as S1, S2 and S3, were partially overlapped, with S3 at the bottom and S1 at the top. From the HRTEM image, we can conclude that both S2 and S3 consisted of three stacked atomic layers, whereas S1 had only one atomic layer. Variations in the spatial periods of the atomic arrays on the surface and in the interlayer distance or thickness of atomic layers were found. For example, the spatial period increased from *a* = 0.22 nm in the center to 1.41*a* (=0.31 nm) near the edge (Fig. 2b,c). The increase in the average spatial period was as high as 9%. The interlayer distance of the BNNSs was also obviously larger than that of bulk boron nitride, as shown in Fig. 4b,c. The interlayer distance increased with decreasing number of layers for the BNNSs. This phenomenon is attributed to the relaxation of surface atoms due to weaker inter-layer interactions with fewer layers.

**Figure 5:**
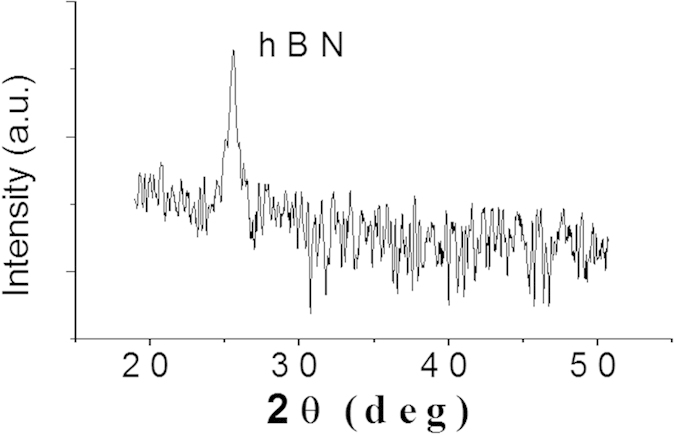
XRD spectrum of BNNSs.

The increase in the interlayer distance for super-thin BNNSs can be confirmed with the data obtained from XRD, wherein a shift of the XRD peak toward a lower diffraction angle was observed. Figure 5 shows a typical XRD pattern for the BNNSs. The high intensity peak centered at 2θ ≈ 25.5° is associated with the hexagonal structure. Thus, the obtained interlayer distance was 0.35 nm for the BNNS according to Bragg’s Law. This value is almost 6% larger than that of bulk BN (*d* = 0.33 nm). Because the bandgap width or energy of III-V nitride materials is normally inversely proportional to the lattice constant^32^, we would expect a 6% decrease in the bandgap width of 2D BNNSs that should result in a red shift in the cutoff wavelength. In fact, the red shift had already been observed in our recent experiments with super-thin BNNS-based deep UV detectors, which exhibited a sharp cutoff wavelength at 250 nm^33^, shifting almost 8% from the cutoff wavelength of 230 nm in bulk h-BN-based photodetectors^34^. All of the obtained results are in good agreement with each other.

### Electrical properties

**Figure 6:**
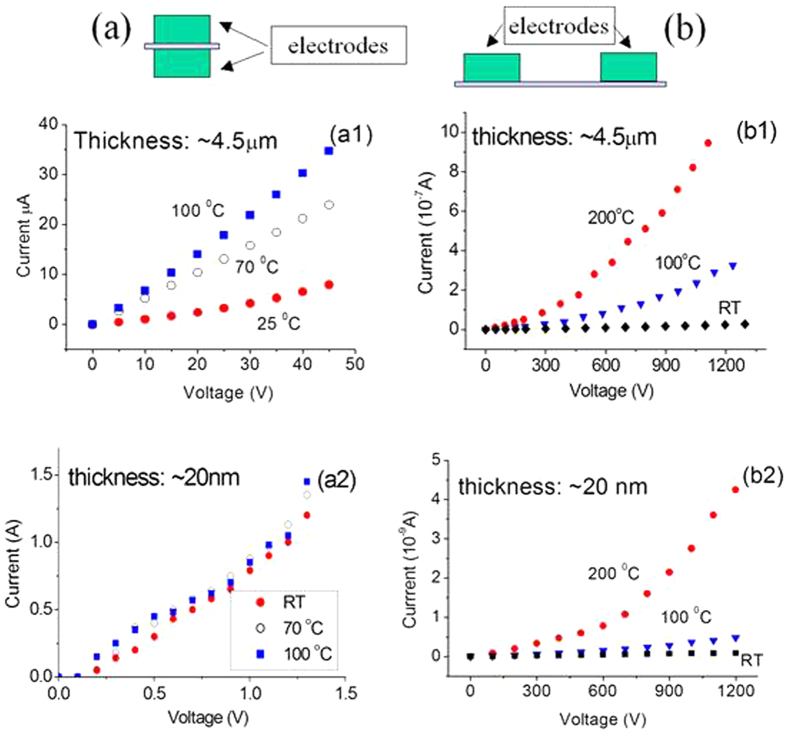
(**a,b**) Two experimental setups for the characterizations of electrical properties of BNNSs. Typical I-V properties of 4.5 μm and 20 nm BNNSs observed in the longitudinal (**a1,a2**) and transverse (**b1,b2**) directions, respectively, at different temperatures.

Characterizations of electrical current-voltage (I-V) properties were also conducted for BNNSs of different thicknesses at different temperatures. The measurements were conducted using a HP - Agilent 6268B Power Supply, a Keithley 6517A electrical meter, and a HEWLETT 34401A electrical meter. Two electrode configurations are shown in Fig. 6a,b. The error for the measurements was approximately 15%. The experimental data (Fig. 6a1) obtained from the setup shown in Fig. 6a indicated that a 225-fold decrease in the thickness of the BNNS membrane (from 4.5 μm to 20 nm) resulted in an approximately five orders of magnitude increase in the electrical current across the BNNS. Spatially non-uniform membranes could have contributed to the phenomenon above, but it would not have been expected to make such a great difference. We believe that the current increase is due to the tunneling effect that usually occurs in the case of super-thin films^35^,^36^.

Using experimental data from Fig. 6a1, where the resistances are inversely proportional to the slopes of curves, and a simple wire model for resistance *R* = *ρl*/*A*, where *l* is the length and *A* is the cross-sectional area, we can estimate the different resistances *R* and resistivities *ρ* (the constant of proportionality) of the BNNSs. The basic parameters are listed in Table 1. The experimental data indicate that the electrical resistivity *ρ* (the inverse of conductivity) of the BNNSs remains almost unchanged for thick BNNSs^36^. However, the resistivity *ρ* decreases sharply with decreasing sheet thickness from 6.7 × 10^6^ Ωm at 4.5 μm to 275 Ωm at 20 nm. Large differences in the resistivity or conductivity coefficients likely indicate that different mechanisms affect the resistances of BNNSs with different thicknesses.

The effect of temperature on the resistivity was clearly visible. Super-thin BNNSs exhibited quasi-metallic properties. Two conclusions can be made after comparing the data listed in Table 1. First, for a sheet thickness of 4.5 μm, the higher the temperature is, the lower the resistance will be. However, there was no obvious temperature effect on the I-V curves of the 20 nm thick BNNSs. Scattering mechanisms may have dominated changes in the I-V curves, as discussed below. Second, for a sheet thickness of approximately 20 nm, electrical breakdown occurred, as shown in Fig. 6a2, where the current increased rapidly at a bias voltage of 1.3 V.

Two mechanisms lead to breakdown: avalanche multiplication and the quantum mechanical tunneling of carriers through the bandgap or barrier height. Neither of the breakdown mechanisms is destructive. Quantum mechanical tunneling of carriers through the barrier height is the dominant breakdown mechanism for metal-semiconductor junctions. This is analogous to tunneling in a highly doped p-n junction, where the energy bandgap is replaced by the barrier height of the material. Using a compact analytical model of band-to-band tunneling in a nanoscale p-i-n diode, Ahmed estimated that the intrinsic region length should be less than 50 nm^37^. The tunneling probability Θ can be calculated from Eq. (1)^38^,^39^:


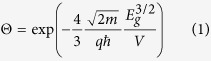


where, the electric potential *V *= *E*_g_/(*qL*). The term *q, m, L*, and *h* are denoted as the electron charge, free electron mass, separation between the two electrodes, and Plank’s constant, respectively. *E*_*g*_ is the barrier height energy and 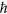 = *h*/(2π).

The tunneling current is obtained based on the values of the carrier charge, velocity and carrier density. The velocity equals to the Richardson velocity, *v*_*R*_, at which, on average, the carriers approach the barrier. The carrier density *J*_*n*_ is proportional to the density of available electrons, *n*, multiplied by the tunneling probability Θ, i.e., 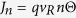. The tunneling current therefore depends exponentially on the barrier height energy to the 3/2 power. Similar measurements were also conducted for the BNNSs in the transverse direction based on the electrode setup illustrated in Fig. 6b. Figure 6b1 show typical I-V characteristics of the 4.5 μm and 20 nm BNNS membranes, respectively. Nonlinearity in the I-V curves was observed and attributed to the polarization effect or cumulative effect of charges. Normally, electrode polarization is due to the migration of some electrons from the metal probe into the samples. As a result, positive charges in the samples migrate towards the metallic probe, thus creating a cation layer between the metallic probe and the samples. The distance between the electrodes was 0.5 mm.

It was found that the I-V curves in Fig. 6a2 could be divided into two areas. At low bias voltages (LV), where the barrier is not severely deformed by the applied electric field, the electric current (I) depends linearly on applied bias V. In the high bias (HV) regime, the electric current becomes nonlinear and increases quickly. Thus, the tunneling process is dominated by field emission tunneling across the barrier. The basic electrical parameters, such as resistances and resistivities as shown in Fig. 6b1 , were obtained, as listed in Table 2. Both samples with different thicknesses at different bias voltages had similar resistivities of approximately 2 × 10^6^ Ωm at room temperature.

Following an increase in temperature from 25 °C to 200 °C, the resistivity decreased from 10^6^ Ωm to 10^4^ Ωm at high bias voltages and to 10^5^ Ωm at low bias voltages. This is very similar to the case in Fig. 6a1 but very different from that shown in Fig. 6a2. It is well known that the resistivity (and conductivity) of BNNSs is actually determined by two factors: the concentration of free carriers available to conduct current and their mobility. There are two basic types of scattering mechanisms that can influence the mobility of electrons and holes: lattice scattering and impurity scattering. Lattice vibrations change carrier mobility with increasing temperature. However, the mobility of the carriers in a semiconductor is also influenced by the presence of charged impurities, and the impurity scattering is caused by crystal defects such as ionized impurities. At lower temperatures, carriers move more slowly, so there is more time for them to interact with charged impurities. As the temperature decreases, the impurity scattering increases, which results in a decrease in carrier mobility; this has the opposite effect as lattice scattering. The total mobility then is the sum of the lattice-scattering mobility and impurity-scattering mobility. Effects of scattering are always present in three-dimensional (3D) bulk materials or in thick films (Fig. 6a1); as a result, the temperature effect on the resistivity/conductivity of such material can be easily identified.

However, in the 2D case, the temperature effect on the resistivity depends on the direction of observation. Lattice vibrations and mobility changes caused by thermal energy in 2D nanosheets only occur in the horizontal/transversal direction. If the electric current were directed along the vertical/longitudinal direction, as shown in Fig. 6a, the scattering caused by temperature changes would not seriously affect the currents (Fig. 6a2). Consequently, the I-V curves remain unchanged when the temperature increases. By contrast, in the case shown in Fig. 6b, wherein the electric current is directed in the horizontal/transversal direction, the scatterings caused by temperature changes unavoidably affect the electrical current, ultimately resulting in variations in the resistivity of the BNNSs, as shown in Fig. 6b1.

## Conclusions

The PLPD technique was used to rapidly synthesize high-quality h-BN nanosheets with a defect-free single crystalline structure, as confirmed by various characterization instruments. The h-BNNSs show zigzag edge reconstructions terminated by boron atoms. The basic mechanism for the BNNS formation is related to thermo-mechanical exfoliation, which is completely different from that for BNNS growth using CVD technique which is physical contact exfoliation process. The obtained experimental data indicate that decreasing of the thickness of BNNSs down to a few atomic layers, not only results in an increase of the spacing between the atoms, i.e., a modified honeycomb crystalline structure of six-membered B_3_-N_3_ hexagons close to the edges, but also results in an increase of the interlayer distance.

The two conclusions made from the electrical property measurement are as follows: (1) the value of the electrical conductivity or resistivity of the super-thin BNNS relies strongly on the direction of observation. The electrical resistivities of the BNNSs decrease sharply when their thickness decreases, when observed from the longitudinal direction. Super-thin BNNSs exhibit good quasi-metal or semiconducting properties. By contrast, when observed from the transversal direction, the electrical resistivities of the BNNSs remain largely unchanged. (2) Temperature effects on the resistivity or conductivity of 3D bulk materials and thick films are inevitable due to scattering. However, in 2D BNNSs, vibrations or mobility due to thermal energy only occur in the horizontal/transversal direction. Therefore, electrical current along vertical/longitudinal direction is not affected by scatterings. Consequently, the resistivity of BNNSs is extremely low, and this value remains nearly constant when the temperature increases.

## Additional Information

**How to cite this article**: Aldalbahi, A. *et al*. Variations in Crystalline Structures and Electrical Properties of Single Crystalline Boron Nitride Nanosheets. *Sci. Rep*. **5**, 16703; doi: 10.1038/srep16703 (2015).

## Figures and Tables

**Table 1:** Basic parameters obtained from the longitudinal setup, as shown in Fig. 6a.

	100 °C	70 °C	25 °C
R_a1_ (Ω)	1.6 × 10^6^	2.1 × 10^6^	6.0 × 10^6^
ρ_a1_ (Ωm)	1.7 × 10^6^	2.3 × 10^6^	6.7 × 10^6^
R_a2_ (Ω)	1.1	1.1	1.1
ρ_a2_ (Ωm)	275	275	275

**Table 2:** Basic parameters obtained from the transverse setup, as shown in Fig. 6b.

	200 °C	100 °C	25 °C
R_b1-HV_ (Ω)	0.63 × 10^9^	1.9 × 10^9^	3.2 × 10^10^
ρ_b1-HV_ (Ωm)	0.028 × 10^6^	0.086 × 10^6^	1.44 × 10^6^
R_b1-LV_ (Ω)	2.6 × 10^9^	9.2 × 10^9^	7.9 × 10^10^
ρ_b1-LV_ (Ωm)	0.12 × 10^6^	0.41 × 10^6^	3.6 × 10^6^
R_b2-HV_ (Ω)	1.5 × 10^11^	1.5 × 10^12^	1.3 × 10^13^
ρ_b2-HV_ (Ωm)	0.03 × 10^6^	0.3 × 10^6^	2.6 × 10^6^
R_b2-LV_ (Ω)	8.1 × 10^11^	4.4 × 10^12^	1 × 310^13^
ρ_b2-LV_ (Ωm)	0.16 ×10^6^	0.8 × 10^6^	2.6 × 10^6^
